# Impedance Variation in a Coaxial Coil Encircling a Metal Tube Adapter

**DOI:** 10.3390/s23198302

**Published:** 2023-10-07

**Authors:** Yao Luo, Xinyi Yang

**Affiliations:** 1 State Key Laboratory of Power Grid Environmental Protection, School of Electrical Engineering and Automation, Wuhan University, Wuhan 430072, China; yangxy@whu.edu.cn; 2 School of Electrical Engineering and Automation, Wuhan University, Wuhan 430072, China

**Keywords:** tube adapter, eddy current, numerical eigenfunctions, Clenshaw–Curtis quadrature

## Abstract

The impedance change in an induction coil surrounding a metal tube adapter is investigated using the truncated region eigenfunction expansion (TREE) method. The conventional TREE method is inapplicable to this problem as a consequence of the numerical overflow of the eigenfunctions of the air–metal multi-subdomain regions. The difficulty is surmounted by a normalization procedure for the numerical eigenfunctions obtained from the 1D finite element method (FEM). An efficient algorithm is devised by the Clenshaw–Curtis quadrature rule for integrals involving the numerical eigenfunctions. The numerical results of the TREE and FEM simulation coincide very well in all cases, and the efficiency of the proposed method is also confirmed.

## 1. Introduction

A tube adapter is a component connecting two tubes of different diameters. The standard analytical method of Dodd and Deeds [1] is unable to investigate the interaction of an induction coil with a metal tube adapter due to the end effects involved in this problem. The truncated region eigenfunction expansion (TREE) method, pioneered by Hannakam and Tepe [2], and developed by Theodoulidis, Kriezis, and Bowler [3,4,5,6,7,8,9] for the modeling of the eddy current nondestructive testing (EC NDT), is capable of analyzing the end effects and establishing analytical models. However, the successful implementation of TREE for the model of end effects depends on the solution of relevant eigenvalue equations, which are transcendental, and complex roots should be determined. Conventionally, the Newton–Raphson algorithm [10,11,12,13] or contour integral based on the Cauchy’s theorem [14,15,16,17] are applied to solve the eigenvalue equations. A novel method based on the Sturm–Liouville theory and Galerkin approach has been proposed recently [18,19,20], which greatly simplifies the process of locating the complex eigenvalues.

**Figure 1:**
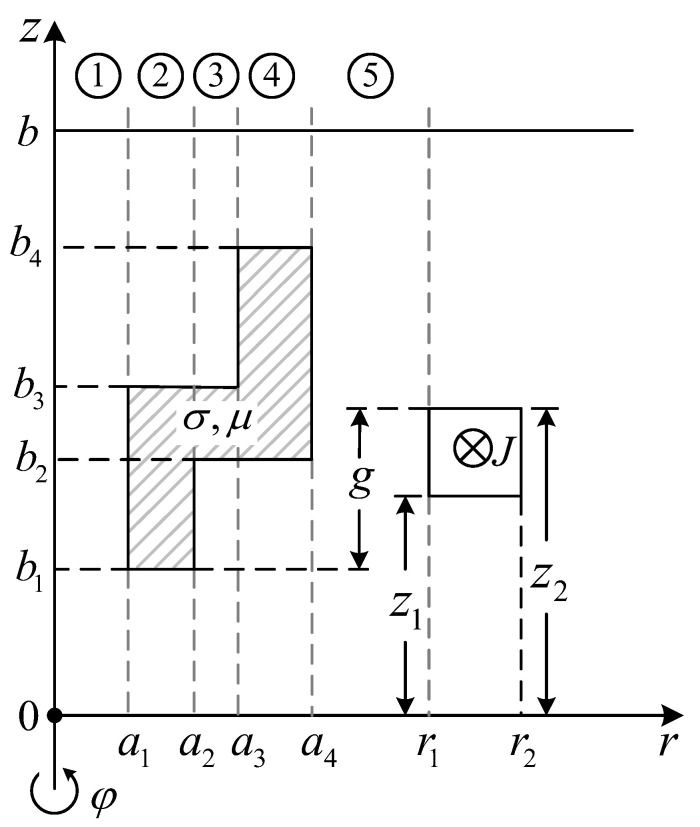
Side view of a metal tube adapter encircled by a coaxial coil.

However, the TREE method has hitherto been available only for problem of the air–metal region of two subdomains. For a problem involving the region of three air–metal subdomains, the source should be decomposed into the odd and even parts, if possible, to reduce the problem to the two subdomains [6,8,21,22,23]. No solutions for the problem involving regions of more subdomains have yet been found in the literature. The difficulty lies in the fact that the symbolic piecewise eigenfunctions for regions of three or more subdomains will become extremely clumsy, and more seriously, they are very prone to numerical overflow with the complex argument, especially when the argument has a relatively large imaginary part. Nevertheless, the issue of numerical overflow should not be superficially ascribed to the multi-subdomain regions but rather to the formally constructed eigenfunctions. By a proper normalization of the eigenfunctions, the overflow could be evaded, and the TREE method should become applicable to problems of multi-subdomain regions. In this work, the normalization of complex eigenfunctions is achieved based on the approach of [19], and a problem including regions of three subdomains (See Figure 1) is solved with TREE.

In Section 2, the TREE solution is given for a metal tube adapter surrounded by a coaxial coil. The permeability of the metal is not restricted to μ_0_. In Section 3, a method successful in dealing with the overflow issue is devised. The numerical eigenfunctions are obtained by the 1D FEM solution of the Sturm–Liouville equations and normalized, and the Clenshaw–Curtis quadrature is applied to the computation of the integrals involving the numerical eigenfunctions. By this strategy, efficient computation of the matrix elements can be contrived. In Section 4, the TREE results are compared with those from the FEM simulation.

## 2. Formulation

**Figure 2:**
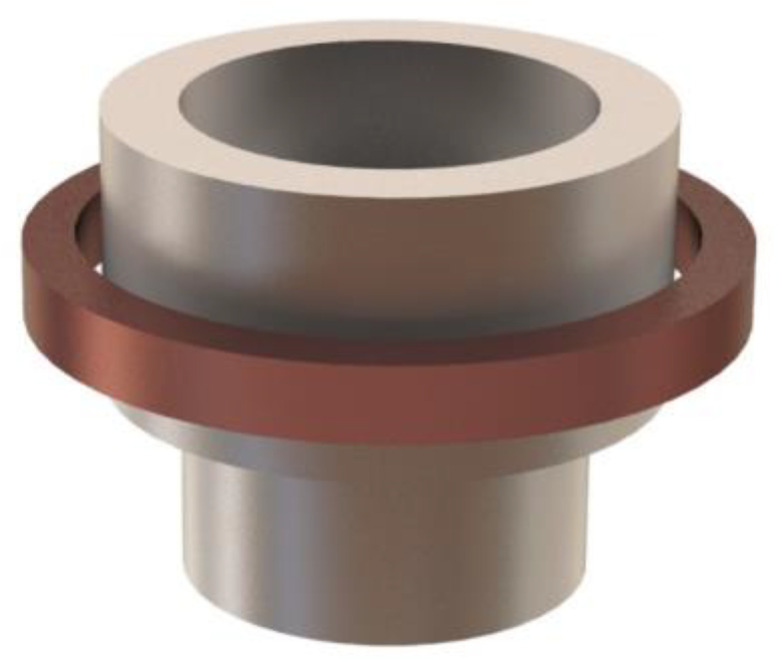
A metal tube adapter encircled by a coaxial coil.

A metal tube adapter of conductivity σ and permeability $\mu =\mu_{r}\mu_{0}$ (*μ_r_* is supposed to be constant) is encircled by a coaxial induction coil excited by a time harmonic current of frequency *ω* and amplitude *I* (See Figure 2). The geometry of the coil and tube adapter is shown in Figure 1. A perfect electric boundary is imposed at *z* = 0 and *z* = *b* to discretize the eigenvalues of this boundary value problem (BVP).

The solution domain is divided into five regions along the *r*-axis (See Figure 1). The vector potentials ***A***_1_ to ***A***_5_ satisfy the Laplace or Helmholtz equations in the corresponding regions:

(1) $$\nabla^{2}A_{1,5}=0$$

(2) $$\nabla^{2}A_{2,3,4}=k^{2}A_{2,3,4}$$

where $k=\sqrt{i\omega \sigma \mu_{0}\mu_{r}}$ is the wavenumber of the metal.

Only the *φ*-component of the vector potential exists due to the axisymmetry of the BVP, i.e., $A=Ae_{\varphi }$, and the vector Laplacian of Equations (1) and (2) is reduced to

(3) $$\nabla_{\varphi }^{2}=\frac{\partial^{2}}{\partial r^{2}}+\frac{1}{r}\frac{\partial }{\partial r}-\frac{1}{r^{2}}+\frac{\partial^{2}}{\partial z^{2}}$$

### 2.1. Vector Potential of the Source Coil

**Figure 3:**
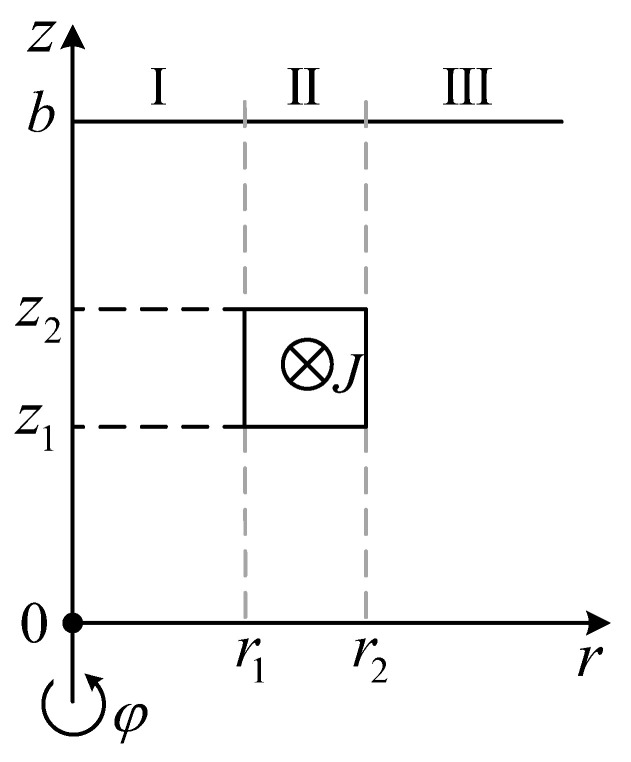
Side view of an isolated coil with truncation boundary.

The formulation of the source vector potential can be obtained by the source expansion of the Poisson equation [24,25]. The vector potential of the coil can be written in the form outlined in Figure 3,

(4a) $$A_{I}\left(r,z\right)=S^{T}\left(z\right)I_{1}\left(\alpha r\right)C_{1}^{\left(e\right)}$$

(4b) $$A_{II}\left(r,z\right)=S^{T}\left(z\right)\left[I_{1}\left(\alpha r\right)C_{2}^{\left(e\right)}+K_{1}\left(\alpha r\right)D_{2}^{\left(e\right)}+V\left(r\right)\right]$$

(4c) $$A_{III}\left(r,z\right)=S^{T}\left(z\right)K_{1}\left(\alpha r\right)D_{3}^{\left(e\right)}$$

Where the source vector **V**(*r*) is

$$V\left(r\right)=\left[\begin{array}{c} v_{1}\left(r\right)\\ v_{2}\left(r\right)\\ \vdots \end{array}\right]$$

with the elements

(5) $$v_{i}\left(r\right)=\kappa_{i}L_{1}\left(\alpha_{i}r\right)$$

where $\alpha_{i}=i\pi /b$, and **L***_n_*(*x*) is the modified Struve function of order *n*, and

(6) $$\kappa_{i}=\frac{2\mu_{0}J}{i\alpha_{i}^{2}}\sin\left[\frac{\alpha_{i}}{2}\left(z_{1}-z_{2}\right)\right]\sin\left[\frac{\alpha_{i}}{2}\left(z_{1}+z_{2}\right)\right]$$

Other matrices and vectors in (4a)–(4c) are

$$\alpha =\left[\begin{array}{ccc} \alpha_{1} & 0 & \cdots \\ 0 & \alpha_{2} & \cdots \\ \vdots & \vdots & \ddots \end{array}\right],\;I_{1}\left(\alpha r\right)=\left[\begin{array}{ccc} I_{1}\left(\alpha_{1}r\right) & 0 & \cdots \\ 0 & I_{1}\left(\alpha_{2}r\right) & \cdots \\ \vdots & \vdots & \ddots \end{array}\right],\;K_{1}\left(\alpha r\right)=\left[\begin{array}{ccc} K_{1}\left(\alpha_{1}r\right) & 0 & \cdots \\ 0 & K_{1}\left(\alpha_{2}r\right) & \cdots \\ \vdots & \vdots & \ddots \end{array}\right],\;S\left(z\right)=\left[\begin{array}{c} \sin\left(\alpha_{1}z\right)\\ \sin\left(\alpha_{2}z\right)\\ \vdots \end{array}\right],$$

where *I_n_*(*x*) and *K_n_*(*x*) are the modified Bessel functions of the first and second kinds of order *n*, respectively, and $C_{1}^{\left(e\right)}$, $C_{2}^{\left(e\right)}$, $D_{2}^{\left(e\right)}$, $D_{3}^{\left(e\right)}$ are the coefficients to be determined. With the interface conditions of *B*_r_ and *H*_z_ at *r* = *r*_1_ and *r* = *r*_2_, the coefficients can be found:

(7a) $$C_{1,i}^{\left(e\right)}=\kappa_{i}\left[\chi \left(\alpha_{i}r_{1}\right)-\chi \left(\alpha_{i}r_{2}\right)\right]$$

(7b) $$C_{2,i}^{\left(e\right)}=-\kappa_{i}\chi \left(\alpha_{i}r_{2}\right)$$

(7c) $$D_{2,i}^{\left(e\right)}=\kappa_{i}\eta \left(\alpha_{i}r_{1}\right)$$

(7d) $$D_{3,i}^{\left(e\right)}=\kappa_{i}\left[\eta \left(\alpha_{i}r_{1}\right)-\eta \left(\alpha_{i}r_{2}\right)\right]$$

where

(8a) $$\chi \left(x\right)=x\left[K_{1}\left(x\right)L_{0}\left(x\right)+K_{0}\left(x\right)L_{1}\left(x\right)\right]$$

(8b) $$\eta \left(x\right)=x\left[I_{1}\left(x\right)L_{0}\left(x\right)-I_{0}\left(x\right)L_{1}\left(x\right)\right]$$

For the function *χ*(*x*) used for the subsequent analysis, it is advisable to adopt an alternative form for the practical evaluations, namely

(9) $$\chi \left(x\right)=\left\{ \begin{array}{l} \frac{x^{2}}{2}\sum_{m=0}^{m_{0}}\frac{{\left(x/2\right)}^{2m}}{\Gamma \left(m+3/2\right)}\left[\frac{K_{1}\left(x\right)}{\Gamma \left(m+3/2\right)}+\frac{xK_{0}\left(x\right)}{2\Gamma \left(m+5/2\right)}\right],x<15\\ 1+\frac{x}{\pi^{2}}\sum_{m=0}^{m_{1}}\frac{\Gamma^{2}\left(m+1/2\right)}{{\left(x/2\right)}^{2m}}\left[\frac{K_{0}\left(x\right)}{m-1/2}-\frac{K_{1}\left(x\right)}{x/2}\right],x\geq 15 \end{array}\right.$$

Expression (9) is obtained by the Maclaurin and asymptotic expansions of **L***_n_*(*x*) [26], and high accuracy can be achieved by setting *m*_0_ = 23 and *m*_1_ = 10, respectively.

### 2.2. Impedance Change in the Coil Encircling the Metal Tube Adapter

The vector potentials in the five regions of Figure 2 are expansible by the separation of variables

(10a) $$A_{1}\left(r,z\right)=S^{T}\left(z\right)I_{1}\left(\alpha r\right)C_{1}$$

(10b) $$A_{2}\left(r,z\right)=F^{T}\left(z\right)\left[I_{1}\left(P_{1}r\right)C_{2}+K_{1}\left(P_{1}r\right)D_{2}\right]$$

(10c) $$A_{3}\left(r,z\right)=G^{T}\left(z\right)\left[I_{1}\left(P_{2}r\right)C_{3}+K_{1}\left(P_{2}r\right)D_{3}\right]$$

(10d) $$A_{4}\left(r,z\right)=H^{T}\left(z\right)\left[I_{1}\left(P_{3}r\right)C_{4}+K_{1}\left(P_{3}r\right)D_{4}\right]$$

(10e) $$A_{5}\left(r,z\right)=S^{T}\left(z\right)\left[I_{1}\left(\alpha r\right)C_{1}^{\left(e\right)}+K_{1}\left(\alpha r\right)D^{\left(s\right)}\right]$$

where **P**_1_, **P**_2_, and **P**_3_ are the eigenvalue matrices of regions 2, 3, and 4, respectively,

$$P_{1}=\left[\begin{array}{ccc} p_{1,1} & 0 & \cdots \\ 0 & p_{1,2} & \cdots \\ \vdots & \vdots & \ddots \end{array}\right],\;P_{2}=\left[\begin{array}{ccc} p_{2,1} & 0 & \cdots \\ 0 & p_{2,2} & \cdots \\ \vdots & \vdots & \ddots \end{array}\right],\;P_{3}=\left[\begin{array}{ccc} p_{3,1} & 0 & \cdots \\ 0 & p_{3,2} & \cdots \\ \vdots & \vdots & \ddots \end{array}\right],$$

and

$$F\left(z\right)=\left[\begin{array}{c} f_{1}\left(p_{1,1},z\right)\\ f_{2}\left(p_{1,2},z\right)\\ \vdots \end{array}\right],\;G\left(z\right)=\left[\begin{array}{c} g_{1}\left(p_{2,1},z\right)\\ g_{2}\left(p_{2,2},z\right)\\ \vdots \end{array}\right],\;H\left(z\right)=\left[\begin{array}{c} h_{1}\left(p_{3,1},z\right)\\ h_{2}\left(p_{3,2},z\right)\\ \vdots \end{array}\right]$$

are the axial eigenfunctions satisfying the relevant Sturm–Liouville equations:

(11a) $$\frac{d^{2}f_{i}\left(z\right)}{dz^{2}}-k_{1}^{2}\left(z\right)f_{i}\left(z\right)=-p_{1,i}^{2}f_{i}\left(z\right),\;f_{i}\left(0\right)=f_{i}\left(b\right)=0$$

(11b) $$\frac{d^{2}g_{i}\left(z\right)}{dz^{2}}-k_{2}^{2}\left(z\right)g_{i}\left(z\right)=-p_{2,i}^{2}g_{i}\left(z\right),\;g_{i}\left(0\right)=g_{i}\left(b\right)=0$$

and

(11c) $$\frac{d^{2}h_{i}\left(z\right)}{dz^{2}}-k_{3}^{2}\left(z\right)h_{i}\left(z\right)=-p_{3,i}^{2}h_{i}\left(z\right),\;h_{i}\left(0\right)=h_{i}\left(b\right)=0$$

with

(12a) $$k_{1}\left(z\right)=\left\{ \begin{array}{l} k,b_{1}\leq z\leq b_{3}\\ 0,\mathrm{others} \end{array}\right.$$

(12b) $$k_{2}\left(z\right)=\left\{ \begin{array}{l} k,b_{2}\leq z\leq b_{3}\\ 0,\mathrm{others} \end{array}\right.$$

and

(12c) $$k_{3}\left(z\right)=\left\{ \begin{array}{l} k,b_{2}\leq z\leq b_{4}\\ 0,\mathrm{others} \end{array}\right.$$

Taking account of the interface conditions of *B_r_* and *H_z_* at *r* = *a*_1_, *r* = *a*_2_, *r* = *a*_3_, and *r* = *a*_4_, the following equations for the coefficients $C_{2}$, $C_{3}$, $C_{4}$, $D_{1}$, $D_{2}$, and $D_{3}$ can be derived

(13a) $$\frac{b}{2}I_{1}\left(\alpha a_{1}\right)C_{1}=T_{1}\left[I_{1}\left(P_{1}a_{1}\right)C_{2}+K_{1}\left(P_{1}a_{1}\right)D_{1}\right]$$

(13b) $$T_{1}^{T}\alpha I_{0}\left(\alpha a_{1}\right)C_{1}=P_{1}\left[I_{0}\left(P_{1}a_{1}\right)C_{2}-K_{0}\left(P_{1}a_{1}\right)D_{2}\right]$$

(13c) $$I_{1}\left(P_{1}a_{2}\right)C_{2}+K_{1}\left(P_{1}a_{2}\right)D_{2}=T_{2}\left[I_{1}\left(P_{2}a_{2}\right)C_{3}+K_{1}\left(P_{2}a_{2}\right)D_{3}\right]$$

(13d) $$T_{2}^{T}P_{1}\left[I_{0}\left(P_{1}a_{2}\right)C_{2}-K_{0}\left(P_{1}a_{2}\right)D_{2}\right]=P_{2}\left[I_{0}\left(P_{2}a_{2}\right)C_{3}-K_{0}\left(P_{2}a_{2}\right)D_{3}\right]$$

(13e) $$I_{1}\left(P_{2}a_{3}\right)C_{3}+K_{1}\left(P_{2}a_{3}\right)D_{3}=T_{3}\left[I_{1}\left(P_{3}a_{3}\right)C_{4}+K_{1}\left(P_{3}a_{3}\right)D_{4}\right]$$

(13f) $$T_{3}^{T}P_{2}\left[I_{0}\left(P_{2}a_{3}\right)C_{3}-K_{0}\left(P_{2}a_{3}\right)D_{3}\right]=P_{3}\left[I_{0}\left(P_{3}a_{3}\right)C_{4}-K_{0}\left(P_{3}a_{3}\right)D_{4}\right]$$

(13g) $$T_{4}\left[I_{1}\left(P_{3}a_{4}\right)C_{4}+K_{1}\left(P_{3}a_{4}\right)D_{4}\right]=\frac{b}{2}\left[I_{1}\left(\alpha a_{4}\right)C_{1}^{\left(e\right)}+K_{1}\left(\alpha a_{4}\right)D^{\left(s\right)}\right]$$

(13h) $$P_{3}\left[I_{0}\left(P_{3}a_{4}\right)C_{4}-K_{0}\left(P_{3}a_{4}\right)D_{4}\right]=T_{4}^{T}\alpha \left[I_{0}\left(\alpha a_{4}\right)C_{1}^{\left(e\right)}-K_{0}\left(\alpha a_{4}\right)D^{\left(s\right)}\right]$$

where

(14) $$T_{1}=\int_{0}^{b}S\left(z\right)F^{T}\left(z\right)dz$$

(15) $$T_{2}=\int_{0}^{b}\frac{1}{\mu_{r}^{\left(1\right)}\left(z\right)}F\left(z\right)G^{T}\left(z\right)dz$$

(16) $$T_{3}=\int_{0}^{b}\frac{1}{\mu_{r}^{\left(2\right)}\left(z\right)}G\left(z\right)H^{T}\left(z\right)dz$$

(17) $$T_{4}=\int_{0}^{b}S\left(z\right)H^{T}\left(z\right)dz$$

In (13a)–(13h), the orthogonalities of the eigenfunctions

(18a) $$\int_{0}^{b}S\left(z\right)S^{T}\left(z\right)dz=\frac{b}{2}I$$

(18b) $$\int_{0}^{b}\frac{1}{\mu_{r}^{\left(1\right)}\left(z\right)}F\left(z\right)F^{T}\left(z\right)dz=I$$

(18c) $$\int_{0}^{b}\frac{1}{\mu_{r}^{\left(2\right)}\left(z\right)}G\left(z\right)G^{T}\left(z\right)dz=I$$

(18d) $$\int_{0}^{b}\frac{1}{\mu_{r}^{\left(3\right)}\left(z\right)}H\left(z\right)H^{T}\left(z\right)dz=I$$

have been adopted, where **I** is the identity matrix, and

(19a) $$\mu_{r}^{\left(1\right)}\left(z\right)=\left\{ \begin{array}{l} \mu_{r},b_{1}\leq z\leq b_{3}\\ 1,\;\mathrm{others} \end{array}\right.$$

(19b) $$\mu_{r}^{\left(2\right)}\left(z\right)=\left\{ \begin{array}{l} \mu_{r},b_{2}\leq z\leq b_{3}\\ 1,\;\mathrm{others} \end{array}\right.$$

(19c) $$\mu_{r}^{\left(3\right)}\left(z\right)=\left\{ \begin{array}{l} \mu_{r},b_{2}\leq z\leq b_{4}\\ 1,\;\mathrm{others} \end{array}\right.$$

The orthonormalization relations of (18b)–(18d) will be expounded in Section 3.

The matrix algebra of (13a)–(13h) yields the equation system

(20) $$\left[\begin{array}{cccc} A_{11} & A_{12} & 0 & 0\\ A_{21} & A_{22} & A_{23} & A_{42}\\ A_{31} & A_{32} & A_{33} & A_{34}\\ 0 & 0 & A_{43} & A_{44} \end{array}\right]\left[\begin{array}{c} C_{2}\\ D_{2}\\ C_{4}\\ D_{4} \end{array}\right]=\left[\begin{array}{c} 0\\ 0\\ 0\\ E \end{array}\right]$$

where

(21a) $$A_{11}=U_{1}I_{1}\left(P_{1}a_{1}\right)-P_{1}I_{0}\left(P_{1}a_{1}\right)$$

(21b) $$A_{12}=U_{1}K_{1}\left(P_{1}a_{1}\right)+P_{1}K_{0}\left(P_{1}a_{1}\right)$$

(21c) $$A_{21}=I_{1}\left(P_{2}a_{3}\right)M_{3}+K_{1}\left(P_{2}a_{3}\right)M_{1}$$

(21d) $$A_{22}=I_{1}\left(P_{2}a_{3}\right)M_{4}+K_{1}\left(P_{2}a_{3}\right)M_{2}$$

(21e) $$A_{23}=-T_{3}I_{1}\left(P_{3}a_{3}\right)$$

(21f) $$A_{24}=-T_{3}K_{1}\left(P_{3}a_{3}\right)$$

(21g) $$A_{31}=T_{3}^{T}P_{2}\left[I_{0}\left(P_{2}a_{3}\right)M_{3}-K_{0}\left(P_{2}a_{3}\right)M_{1}\right]$$

(21h) $$A_{32}=T_{3}^{T}P_{2}\left[I_{0}\left(P_{2}a_{3}\right)M_{4}-K_{0}\left(P_{2}a_{3}\right)M_{2}\right]$$

(21i) $$A_{33}=-P_{3}I_{0}\left(P_{3}a_{3}\right)$$

(21j) $$A_{34}=P_{3}K_{0}\left(P_{3}a_{3}\right)$$

(21k) $$A_{43}=U_{2}I_{1}\left(P_{3}a_{4}\right)+P_{3}I_{0}\left(P_{3}a_{4}\right)$$

(21l) $$A_{44}=U_{2}K_{1}\left(P_{3}a_{4}\right)-P_{3}K_{0}\left(P_{3}a_{4}\right)$$

(21m) $$E=T_{4}^{T}\alpha \left[I_{0}\left(\alpha a_{4}\right)+K_{0}\left(\alpha a_{4}\right)K_{1}^{-1}\left(\alpha a_{4}\right)I_{1}\left(\alpha a_{4}\right)\right]C_{1}^{\left(e\right)}$$

with

(22a) $$U_{1}=\frac{2}{b}T_{1}^{T}\alpha I_{0}\left(\alpha a_{1}\right)I_{1}^{-1}\left(\alpha a_{1}\right)T_{1}$$

(22b) $$U_{2}=\frac{2}{b}T_{4}^{T}\alpha K_{0}\left(\alpha a_{4}\right)K_{1}^{-1}\left(\alpha a_{4}\right)T_{4}$$

(22c) $$M_{1}=a_{2}\left[X_{1}I_{1}\left(P_{1}a_{2}\right)-X_{2}I_{0}\left(P_{1}a_{2}\right)\right]$$

(22d) $$M_{2}=a_{2}\left[X_{1}K_{1}\left(P_{1}a_{2}\right)+X_{2}K_{0}\left(P_{1}a_{2}\right)\right]$$

(22e) $$M_{3}=a_{2}\left[X_{3}I_{1}\left(P_{1}a_{2}\right)+X_{4}I_{0}\left(P_{1}a_{2}\right)\right]$$

(22f) $$M_{4}=a_{2}\left[X_{3}K_{1}\left(P_{1}a_{2}\right)-X_{4}K_{0}\left(P_{1}a_{2}\right)\right]$$

(22g) $$X_{1}=P_{2}I_{0}\left(P_{2}a_{2}\right)T_{2}^{-1}$$

(22h) $$X_{2}=I_{1}\left(P_{2}a_{2}\right)T_{2}^{T}P_{1}$$

(22i) $$X_{3}=P_{2}K_{0}\left(P_{2}a_{2}\right)T_{2}^{-1}$$

(22j) $$X_{4}=K_{1}\left(P_{2}a_{2}\right)T_{2}^{T}P_{1}$$

Solving Equation (20) will give the coefficients **C**_2_, **D**_2_, **C**_4_, and **D**_4_, and other coefficients can be found by

(23) $$C_{1}=\frac{2}{b}I_{1}^{-1}\left(\alpha a_{1}\right)T_{1}\left[I_{1}\left(P_{1}a_{1}\right)C_{2}+K_{1}\left(P_{1}a_{1}\right)D_{2}\right]$$

(24) $$C_{3}=M_{3}C_{2}+M_{4}D_{2}$$

(25) $$D_{3}=M_{1}C_{2}+M_{2}D_{2}$$

The coefficient required for the calculation of ΔZ is

(26) $$D^{\left(s\right)}=K_{1}^{-1}\left(\alpha a_{4}\right)\left\{-I_{1}\left(\alpha a_{4}\right)C_{1}^{\left(e\right)}+\frac{2}{b}T_{4}\left[I_{1}\left(P_{3}a_{4}\right)C_{4}+K_{1}\left(P_{3}a_{4}\right)D_{4}\right]\right\}$$

Accordingly, the coil impedance variation is given by

(27) $$\begin{array}{l} \Delta Z=\frac{i\omega }{I^{2}}\int_{V}A^{\left(s\right)}\cdot JdV\\ =\frac{\pi^{2}i\omega N^{2}}{{\left(r_{2}-r_{1}\right)}^{2}{\left(z_{2}-z_{1}\right)}^{2}}\sum_{n=1}^{\infty }\frac{\cos\left(\alpha_{n}z_{1}\right)-\cos\left(\alpha_{n}z_{2}\right)}{\alpha_{n}^{3}}\left[\chi \left(\alpha_{n}r_{2}\right)-\chi \left(\alpha_{n}r_{1}\right)\right]d_{n}^{\left(s\right)} \end{array}$$

where the current density *J* has been omitted (letting *J* = 1) to simplify the expression.

## 3. Eigenfunctions and the Associated Integrals of the Multi-Subdomain Regions

In the conventional TREE models, symbolic piecewise eigenfunctions are used for the air–metal multi-subdomain regions. With this approach, the TREE method is restricted to the two-subdomain problems (apart from certain problems of three subdomains). For problems involving air–metal regions of more subdomains, the overflow of the explicit eigenfunctions is inevitable, which raises serious difficulties in the numerical evaluations. Therefore, the eigenfunctions of (11a)–(11c) cannot be treated by the conventional TREE method.

In [18,19,20], the eigenvalue problem of (11a)–(11c) is reformulated in terms of a Sturm–Liouville problem. In accordance with [18,19,20], the eigenvalues of (11a) can be obtained by the solution of a generalized eigenvalue equation

(28) $$KU_{i}=p_{1,i}^{2}WU_{i}$$

where **K** is the stiffness matrix with the elements

(29) $$K_{mn}=\int_{0}^{b}\frac{1}{\mu_{r}^{\left(1\right)}\left(z\right)}[\frac{d\varphi_{m}\left(z\right)}{dz}\frac{d\varphi_{n}\left(z\right)}{dz}+k_{1}^{2}\left(z\right)\varphi_{m}\left(z\right)\varphi_{n}\left(z\right)]dz$$

and **W** is the damping matrix of the elements

(30) $$W_{mn}=\int_{0}^{b}\frac{\varphi_{m}\left(z\right)\varphi_{n}\left(z\right)}{\mu_{r}^{\left(1\right)}\left(z\right)}dz$$

where *φ_m_* and *φ_n_* are the FEM functions consisting of the Lagrange polynomials defined on the reference interval −1≤ξ≤1 (the shape functions).

A sparse matrix **K** will be generated from the FEM basis. Hence, Equation (28) can be solved by an efficient algorithm, such as Arnoldi iteration [27]. This solution provides both the eigenvalues *p*_1*,i*_ and the eigenvectors **U***_i_*, which are the discrete eigenfunctions $f_{i}\left(z\right)$. Moreover, denoting

(31) $$U={\left[U_{1},U_{2},\ldots \right]}^{T}$$

and by virtue of the vector normalization

(32) $$U^{\prime }=\frac{U}{\sqrt{\mathrm{diag}\left(UWU^{T}\right)}}$$

the eigenfunction normalization

(33) $$\int_{0}^{b}\frac{1}{\mu_{r}^{\left(1\right)}}f_{i}^{2}\left(z\right)dz=1$$

can be established automatically. Equations (32) and (33) can be validated by inspecting the diagonal entries of $UWU^{T}$ and taking Equation (30) into account. Consequently, the orthonormality of (18b)–(18d) can be established.

The requirement of the accurate and efficient algorithm leads to the choice of high order Lagrange polynomials for the FEM basis. Here, we choose the cubic Lagrange polynomials

(34) $$\left\{ \begin{array}{l} N_{0}\left(\xi \right)=-\frac{1}{16}\left(\xi -1\right)\left(3\xi -1\right)\left(3\xi +1\right)\\ N_{1}\left(\xi \right)=\frac{9}{16}\left(\xi -1\right)\left(\xi +1\right)\left(3\xi -1\right)\\ N_{2}\left(\xi \right)=-\frac{9}{16}\left(\xi -1\right)\left(\xi +1\right)\left(3\xi +1\right)\\ N_{3}\left(\xi \right)=\frac{1}{16}\left(\xi +1\right)\left(3\xi -1\right)\left(3\xi +1\right) \end{array}\right.$$

The cubic interpolation of the eigenfunction is

(35) $$f_{i}\left(z\right)=\sum_{e=0}^{3}u_{l+e}^{\prime }N_{e}\left(z\right)$$

where $u_{l+e}^{\prime }$ is the successive four entries of $U_{i}^{\prime }$, and *N_e_*(*z*) is obtained by (34) with the change in the variable

(36) $$\xi =\frac{2z-z_{a}-z_{b}}{z_{b}-z_{a}}$$

where *z_a_* and *z_b_* are the mesh nodes corresponding to the reference interval. The numerical overflow of $f_{i}\left(z\right)$ is eliminated by this procedure. They are consequently well adapted for the subsequent integral computation. Furthermore, it appears to be very effective to evaluate directly the integrals (14)–(17) with the Clenshaw–Curtis quadrature, which is quoted here for completeness [28,29,30]

(37) $$\int_{-1}^{1}f\left(x\right)dx=\sum_{k=0}^{n}w_{k}f\left(x_{k}\right)$$

where the weights *w_k_* are given by

(38) $$w_{k}=\frac{g_{k}}{n}\left(1-\sum_{j=1}^{\left\lfloor n/2\right\rfloor }\frac{b_{j}}{4j^{2}-1}\cos\left(2jk\pi /n\right)\right)$$

and the quadrature nodes are

(39) $$x_{k}=\cos\left(\frac{k\pi }{n}\right),k=0,1,\ldots ,n$$

with

(40) $$g_{k}=\left\{ \begin{array}{l} 1\;,\;k=0,n\\ 2\;,\;\mathrm{otherwise} \end{array}\right.$$

(41) $$b_{j}=\left\{ \begin{array}{l} 1\;,\;j=\frac{1}{2}n\\ 2\;,\;\mathrm{otherwise} \end{array}\right.$$

It follows from Equations (37)–(41) that the matrix elements of **T**_1_ can be computed by

(42) $$\begin{array}{l} T_{1,ij}=\int_{0}^{b}\sin\left(\alpha_{i}z\right)f_{j}\left(z\right)dz\\ =\frac{b}{2}\int_{-1}^{1}\sin\left[\frac{b}{2}\alpha_{i}\left(1+x\right)\right]f_{j}\left[\frac{b}{2}\left(1+x\right)\right]dx\\ =\frac{b}{2}\sum_{k=0}^{n}w_{k}\sin\left(\alpha_{i}z_{k}\right)f_{j}\left(z_{k}\right) \end{array}$$

where

(43) $$z_{k}=\frac{b}{2}\left(1+x_{k}\right)$$

The matrix elements of **T**_2_ are likewise given by

(44) $$\begin{array}{l} T_{2,ij}=\int_{0}^{b}\frac{1}{\mu_{r}^{\left(1\right)}}f_{i}\left(z\right)g_{j}\left(z\right)dz\\ =\int_{0}^{b_{1}}f_{i}\left(z\right)g_{j}\left(z\right)dz+\frac{1}{\mu_{r}}\int_{b_{1}}^{b_{3}}f_{i}\left(z\right)g_{j}\left(z\right)dz+\int_{b_{3}}^{b}f_{i}\left(z\right)g_{j}\left(z\right)dz\\ =\frac{b_{1}}{2}\sum_{k=0}^{n}w_{k}f_{i}\left(z_{k}^{\left(1\right)}\right)g_{j}\left(z_{k}^{\left(1\right)}\right)+\frac{b_{3}-b_{1}}{2}\sum_{k=0}^{n}w_{k}f_{i}\left(z_{k}^{\left(2\right)}\right)g_{j}\left(z_{k}^{\left(2\right)}\right)+\frac{b-b_{3}}{2}\sum_{k=0}^{n}w_{k}f_{i}\left(z_{k}^{\left(3\right)}\right)g_{j}\left(z_{k}^{\left(3\right)}\right) \end{array}$$

where

(45) $$z_{k}^{\left(1\right)}=\frac{b_{1}}{2}\left(1+x_{k}\right),z_{k}^{\left(2\right)}=\frac{b_{3}+b_{1}}{2}+\frac{b_{3}-b_{1}}{2}x_{k},z_{k}^{\left(3\right)}=\frac{b+b_{3}}{2}+\frac{b-b_{3}}{2}x_{k}$$

**Figure 4:**
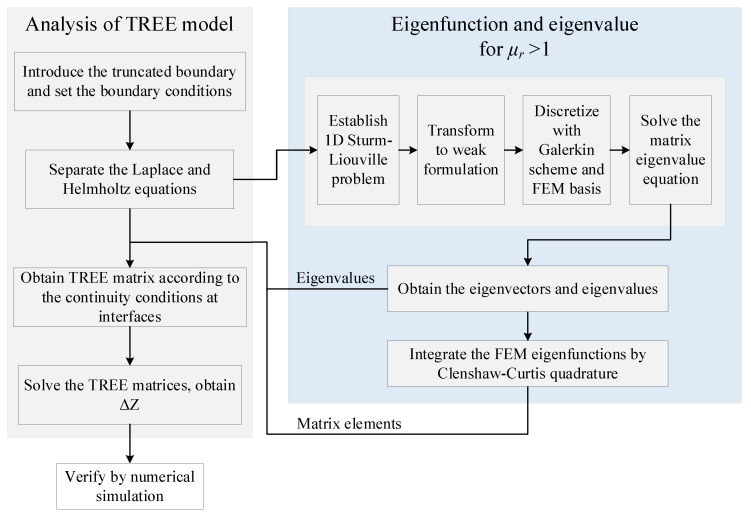
Flowchart of the TREE method enhanced by 1D FEM.

The same analysis is also applicable to the matrix elements of **T**_3_ and **T**_4_. A flowchart is provided in Figure 4 to present the process of the novel approach.

## 4. Numerical Validation

**Figure 5:**
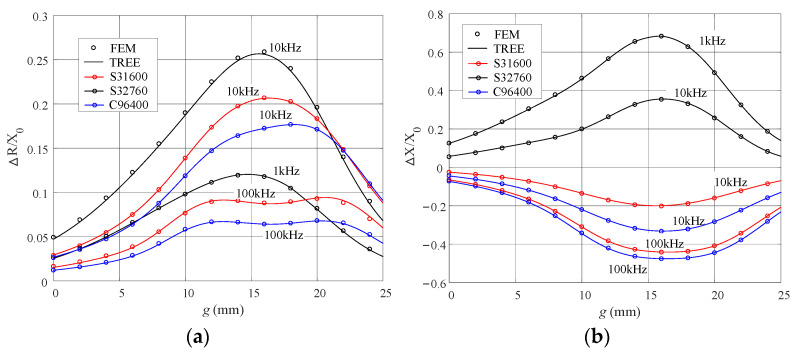
Normalized impedance variations with the abscissa representing the parameter *g*. (**a**) The resistance variation. (**b**) The reactance variation.

The proposed method will be verified with the parameters of the metal tube adapter and the induction coil given in Table 1, Table 2 and Table 3. The nonmagnetic alloy UNS (Unified Numbering System) C96400 (70-30 Copper-Nickel) and the magnetic stainless steels S31600 (austenitic) and S32760 (super duplex) [31] are used for the numerical validation. The coil impedance variations are calculated and plotted for these metal materials with different coil positions. The TREE results are compared with those from the FEM simulation of Comsol Multiphysics^®^(COMSOL Inc., Stockholm, Sweden), shown in Figure 5, where the theoretical and FEM data are denoted by solid lines and circles, respectively. The reactance of the isolated induction coil is *X*_0_ = *ωL*_0_, with *L*_0_ = 4.104132 mH, which can be found by the method such as in [32].

**Figure 6:**
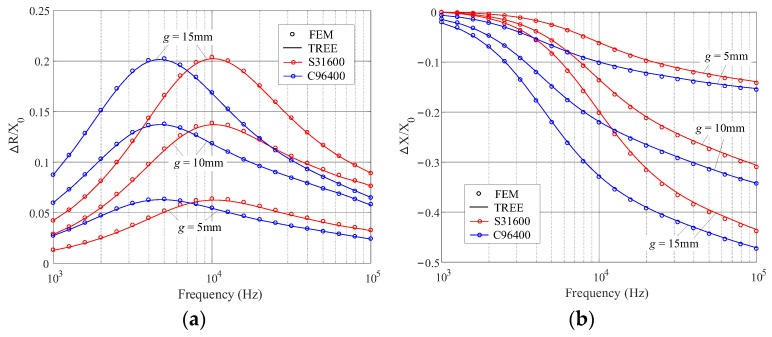
Normalized impedance variations with the abscissa representing the frequency. The alloys are C96400 and S31600. (**a**) The resistance variation. (**b**) The reactance variation.

**Figure 7:**
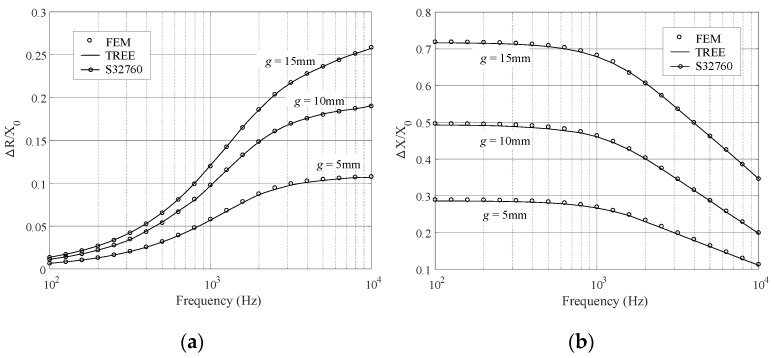
Normalized impedance variations with the abscissa representing the frequency. The alloy is S32760. (**a**) The resistance variation. (**b**) The reactance variation.

Further calculations are carried out for the coil impedance variation with respect to the frequencies. For the alloys of lower *μ_r_* (C96400 and S31600), the calculation frequency ranges from 1 kHz to 100 kHz, for higher *μ_r_* (S32760), the frequency interval [100 Hz, 10 kHz] is chosen. The results are shown in Figure 6 and Figure 7, where the TREE data are plotted by solid lines in connection with the circles representing the data of the FEM simulation. Other parameters are referred to in Table 2 and Table 3.

Very good agreement is obtained between the TREE and FEM results in the numerical comparisons. The calculations were implemented on a personal computer of a 4.2 GHz processor (Intel^®^ Core i7-7700K) and 16 GB RAM. Additional algorithm details are shown in Table 4, where the frequencies, summation terms (matrix size), mesh elements, and quadrature nodes used in the computation are listed. The execution time of the eigenvalue and eigenfunction computation and the total execution time of the TREE evaluation are also provided. No more than 1.5 s (including the time consumed by the calculation of eigenvalues and eigenfunctions) are needed for a TREE evaluation. The satisfactory algorithm efficiency provides evidence for this.

## 5. Conclusions

The interaction of an eddy current coil with a metal tube adapter has been investigated using the TREE method. The numerical overflow for symbolic eigenfunctions of air–metal multi-subdomain regions has been removed via the normalization of the eigenvectors, and a satisfactory computational speed was achieved using the Clenshaw–Curtis quadrature rule applied to the integrals associated with the numerical eigenfunctions. The calculation accuracy has been verified by the numerical comparisons, and the efficiency of our approach has also been confirmed. Considerable potential has been shown for the development of new analytical models with the aid of the proposed approach.

## Figures and Tables

**Table 1:** Metals used for the tube adapter.

Metal (UNS)	Conductivity σ (MS/m)	Relative Permeability *μ_r_*
C96400	2.9	1
S31600	1.33	1.02
S32760	1.25	29

**Table 2:** Geometry of the metal tube adapter.

Parameter		Parameter	
*a*_1_ (mm)	5	*b*_1_ (mm)	40
*a*_2_ (mm)	8	*b*_2_ (mm)	48
*a*_3_ (mm)	11	*b*_3_ (mm)	51
*a*_4_ (mm)	14	*b*_4_ (mm)	59
*b* (mm)	100		

**Table 3:** Parameters of the induction coil.

Parameter	
Inner radius *r*_1_ (mm)	15
Outer radius *r*_2_ (mm)	18
Axial length *z*_2_ *− z*_1_ (mm)	6
Number of turns	300

**Table 4:** Computation configuration and execution time of TREE method.

Metal (UNS)	Frequency	Summation Terms	Quadrature Nodes	Mesh Elements	Execution Time of Eigenvalue and Eigenfunction Computation	Total Execution Time
S31600	10 kHz	30	80	510	0.19 s	0.55 s
	100 kHz	40	80	510	0.26 s	0.73 s
S32760	1 kHz	55	80	510	0.36 s	1.00 s
	10 kHz	70	90	510	0.54 s	1.30 s
C96400	10 kHz	30	80	510	0.19 s	0.56 s
	100 kHz	50	80	510	0.34 s	0.90 s

## Data Availability

Data sharing not applicable.
